# Triptans Use for Migraine Headache among Nonelderly Adults with Cardiovascular Risk

**DOI:** 10.1155/2016/8538101

**Published:** 2016-08-17

**Authors:** Monira Alwhaibi, Arijita Deb, Usha Sambamoorthi

**Affiliations:** School of Pharmacy, Department of Pharmaceutical Systems and Policy, West Virginia University, Morgantown, WV 26506, USA

## Abstract

*Objective*. To examine the association between the cardiovascular (CV) risk factors and triptans use among adults with migraine.* Methods*. A retrospective cross-sectional study design was used. Data were derived from 2009–2013 Medical Expenditure Panel Survey (MEPS). The study sample consisted of adults (age > 21 years) with migraine headache (*N* = 1,652). Multivariable logistic regression was used to examine the relationship between CV risk factors and triptans use.* Results*. Overall, 21% adults with migraine headache used triptans. Nearly two-thirds (61%) of adults with migraine had at least one CV risk factor. A significantly lower percentage of adults with CV risk (18.1%) used triptans compared to those without CV risk factors (25.5%). After controlling for demographic, socioeconomic status, access to care, and health status, adults with no CV risk factors were more likely to use triptans as compared to those with one CV risk factor (AOR = 1.83, 95% CI = 1.17–2.87). There were no statistically significant differences in triptans use between those with two or more CV risk factors and those with one CV risk factor.* Conclusion*. An overwhelming majority of adults with migraine had a contraindication to triptans based on their CV risk factors. The use of triptans among adults with migraine and multiple CV risk factors warrants further investigation.

## 1. Introduction

Migraine headache is a chronic neurovascular and disabling condition that affects nearly 16.6% of adults in the United States (US) [1]. The World Health Organization (WHO) ranked migraine headache as one of the top 20 diseases causing disability [2]. Migraine is associated with poor health-related quality of life (HRQoL), missed work day [3, 4], and high economic burden [5–7]. Migraine is a treatable condition and triptans are the only US Food and Drug Administration (FDA) approved pharmacological therapies for the management of acute episodes of moderate to severe migraine headaches [8, 9].

Triptans are, however, contraindicated in patients with cardiovascular disease (CVD) and should be used with caution in patients with cardiovascular (CV) risk factors (i.e., diabetes, hypertension, hyperlipidemia, obesity, and smoking). Little is known about the rates of triptans use among adults with CV risk factors. Only the American Migraine Prevalence and Prevention (AMPP) study examined triptans use among adults with CV risk factors [10]. This study found that triptans are less likely to be used by individuals with diabetes, hypertension, and smokers as compared to those without diabetes and hypertension and those who are nonsmokers. Furthermore, this study did not include obesity as a CV risk factor. It is well documented that obesity is one of the important CV risk factors [11, 12]. Furthermore, triptans use patterns among adults with multiple CV risk factors are unknown. Such knowledge is critical for clinical practice and to reduce harms associated with triptans use among adults with multiple CV risk factors. Therefore, this study examined the association between CV risk factors and triptans use among adults with migraine headache using a nationally representative sample from the Medical Expenditure Panel Survey (MEPS).

## 2. Methods

### 2.1. Study Design

A retrospective cross-sectional study design was used. Data were extracted from the full year consolidated data files, medical conditions files, and the prescribed medicines files of the MEPS for the years 2009, 2011, and 2013. MEPS is a nationally representative survey of the US noninstitutionalized civilian population [13]. The Household Component (HC) of MEPS collects information on the demographic characteristics, medical conditions, health status, utilization of healthcare services, charges and payments, access to care, health insurance coverage, income, education, and employment on all household members. The medical conditions file of MEPS contains information on medical conditions of the respondents based on the verbatim text and these texts are converted into International Classification of Diseases, Ninth Revision, and Clinical Modification (ICD-9-CM) codes by professional coders. These codes are then classified into clinically homogenous condition codes known as CCS codes. The event-level prescribed medications file contains detailed records of prescribed medicines such as the national drug code (NDC), medicine name, therapeutic class based on Multum Lexicon classification, sources of payment, and fill date. Alternate years of data were used to avoid entering overlapping panels.

### 2.2. Study Population

The study sample is comprised of adults, aged 22–64 years old with a diagnosis of migraine headache (ICD-9-CM code = 346.XX) and who were alive during the calendar years. Our analytical sample consisted of 1,652 adults with migraine headache.

## 3. Measures

### 3.1. Dependent Variable: Use of Triptans Medications

Triptans medications were derived from prescription drug files. In the MEPS, prescription drugs are linked to the Multum Lexicon database. We used the Multum therapeutic class subcategory code (TC1S1_1) which provides information about antimigraine drug class. Antimigraine drug class code of 193 contains only triptans (e.g., sumatriptan, naratriptan).

### 3.2. Key Independent Variable: CV Risk Factors

CV risk factors included diabetes (CCS codes 49, 50 from medical conditions file or an affirmative response to a question about diagnosed diabetes), hypertension (CCS codes 98, 99 from medical conditions file or an affirmative response to a question about diagnosed high blood pressure), hyperlipidemia (CCS code 53), current smoking status (yes/no), and obesity. Obesity was defined using the Body Mass Index (BMI) values and adults with BMI > 30.0 were considered as obese. Current smoking status was extracted from affirmative responses of adult self-administered questionnaire that queried whether or not the respondents currently smoke.

### 3.3. Other Independent Variables

Demographics included gender, age in years (22–39, 40–49, and 50–64), race/ethnicity (White, African American, Latino, and others), marital status (married, widowed, separated/divorced, and never married), and the region of residence (northeast, midwest, south, and west). Socioeconomic characteristics included education (less than high school, high school, and above high school), health insurance (public, private, and uninsured), medication insurance (insured, uninsured), employment status, and poverty status. Poverty status was defined using family income in relation to the federal poverty line (FPL) and classified as poor (<100% FPL), near poor (100% to <200% FPL), middle income (200% to <400% FPL), and high income (≥400% FPL). Other independent variables included moderate to vigorous physical activity (3 times a week or more/less than 3 times a week) and perceived physical health (excellent/very good, good, and fair/poor).

### 3.4. Statistical Analysis

The unadjusted associations between triptans use and independent variables were examined by chi-square tests. Multivariable logistic regressions were used to examine the association between CV risk factors and triptans use after adjusting for sex, race/ethnicity, age, marital status, education, employment, poverty status, health insurance coverage, prescription insurance coverage, health status, physical activity, and region. Parameter estimates from the logistic regression were transformed into adjusted odds ratio (AOR) and their corresponding 95% confidence intervals were reported. We present the association between CV risk factors and triptans use by using “no CV risk” as well as “one CV risk factor” as the reference group. To account for the complex design of MEPS, we conducted all analyses with survey procedures in SAS 9.4 (SAS Institute Inc., Cary, NC).

## 4. Results

### 4.1. Description of the Study Sample

Table 1 displays the characteristics of the study sample (*n* = 1,652). Our study sample of adults with migraine consisted of 75.8% women, 73.6% white, and nearly 43% in the age group 22–39 years. About 73% of the study population had prescription drug coverage. Nearly 61% of adults with migraine had at least one CV risk factor with 29% having only one CV risk factor, 20.3% having two CV risk factors, and 11.5% having three or more risk factors.

### 4.2. Triptans Use and CV Risk Factors

Table 1 also describes triptans use by subjects' characteristics. Triptans use was reported by 21% of adults in our sample. We found that triptans use was reported by a significantly lower percentage of those with CV risk factors (18.1%) as compared to those without CV risk factors (25.5%). When examined by the number of CV risk factors, 16.4% of adults with only one CV risk factor, 22.3% of adults with two CV risk factors, and 14.9% of adults with 3 or more CV risk factors reported use of triptans.

In the adjusted analysis, when “no CV risk factors” were used as the reference group, adults with cardiovascular risk factors were significantly less likely to use triptans as compared to adults without cardiovascular risk (one CV risk factor: AOR = 0.55, 95% CI = 0.35, 0.86; three or more CV risk factors: AOR = 0.36, 95% CI = 0.20, 0.66) (Table 2).

When “one CV risk factor” was used as the reference group, we found that adults without any CV risk factors were significantly more likely to use triptans (AOR, 95% CI). However, we observed that adults with migraine and multiple cardiovascular risk factors did not differ significantly in triptans use as compared to adults with only one CV risk factor (two CV risk factors: AOR = 1.27, 95% CI = 0.74, 2.17; three or more CV risk factors: AOR = 0.66, 95% CI = 0.35, 1.25) (Table 2).

## 5. Discussion

The present study examined the relationship between CV risk factors and triptans use among adults with migraine by comparing those with and without CV risk factors. We found a high prevalence of CV risk factors among adults with migraine with 61% of them reporting at least one cardiovascular risk factor and 31% reporting more than one cardiovascular risk factors. This is consistent with published research in which the risk of CV risk factors among adults with migraine is higher than those without migraine [14, 15]. Bigal et al. reported that the prevalence rate of CV risk factors such as diabetes (12.6%), hypertension (33.1%), hyperlipidemia (32.7%), and smoking (15.8%) was significantly higher among adults with migraine as compared to adults without migraine [10].

The rate of triptan use among those without CV risk factors (25.5%) found in our study sample of adults with migraine is somewhat higher than those found in the published literature. The AMPP study reported that triptans use among those with no CV risk factors was 18.3% [10]. The difference in rates of triptans use may be due to differences in study population and the definition of CV risk factors. AMPP study considered diabetes, hypertension, hyperlipidemia, and current smoking as CV risk factors and included adults over age 64 years. However, our study additionally included obesity as a CV risk factor and restricted our sample to working age adults aged between 18 and 64 years.

We observed statistically significant differences in triptans use by CV risk factors. Our findings revealed that adults with migraine who also had CV risk factors were significantly less likely to use triptans as compared to adults with migraine without any CV risk factors. However, there were no significant differences in triptans use among adults with multiple CV risk factors as compared to adults with only one CV risk factor. FDA's evidence based clinical practice guidelines and consensus statements recommend that healthcare providers should prescribe triptans with caution to patients with CV risk factors [16, 17]. In addition, FDA recommends a cardiac evaluation to those with multiple risk CV risk factors. Previous research has shown that some headache specialists and family practitioners prescribe triptans to patients regardless of the CV risk factors profile and there is a lack of consensus in terms of when triptan should be avoided due to cardiac contraindications and when a cardiac evaluation should be done prior to prescribing a triptan [18]. Therefore, similar rates of triptans use among adults with migraine and multiple cardiac contraindications are concerning and warrant further investigation into the current clinical practice patterns.

The study strength lies in examining triptans use among a nationally representative sample of adults with migraine headache. This study has included all of the CV risk factors and controlled for many confounders. Limitations of this study include lack of measure for severity of migraine headache, patient's attitudes, and preferences toward using prescription drugs.

## 6. Conclusion

There is a high prevalence of CV risk factors that are considered as contraindications to triptans use among adults with migraine and a sizable proportion of adults with migraine who have CV contraindications use triptans for migraine headache. Triptans use, particularly among those with multiple CV risk factors, is concerning and future studies should investigate why triptans are prescribed for these adults and whether such prescriptions are accompanied by systematic and rigorous cardiac evaluations.

## Figures and Tables

**Table 1:** Description of the study sample and number and weighted percent with triptans use adults with migraine headache Medical Expenditure Panel Survey, 2009–2013.

	Total sample		Triptans use		Sig.
	*N*	Wt.%	*N*	Wt.%	
*All*	*1,652*	*100.0*	*292*	*21%*	

*Gender*					*∗*
Women	1,299	75.8	247	22.7	
Men	353	24.2	45	15.4	

*Race/ethnicity*					*∗∗∗*
White	866	73.6	193	24.2	
African American	303	9.4	43	13.3	
Latino	357	11.1	42	11.5	
Others	126	5.8	14	11.1	

*Age in years*					
22–39 years	720	42.9	113	18.7	
40–49 years	468	27.3	77	19.5	
50–64 years	464	29.8	102	25.6	

*Marital status*					
Married	824	55.0	159	22.8	
Widowed/separated/divorced	403	21.5	76	22.9	
Never married	425	23.5	57	15.1	

*Education level*					*∗*
LT HS	263	12.1	29	12.9	
HS	421	19.4	60	17.3	
>HS	957	68.0	202	23.4	

*Employment*					
Employed	1,170	76.0	191	20.6	
Not employed	481	24.0	101	22.3	

*Poverty status*					*∗*
Poor	349	13.1	53	17.9	
Near poor	366	16.5	55	15.1	
Middle income	483	31.0	80	18.8	
High income	454	39.3	104	26.2	

*Health insurance*					*∗∗*
Private	1,048	75.6	199	22.5	
Public	339	12.8	66	20.9	
Uninsured	265	11.5	27	10.9	

*Medication insurance *					*∗∗∗*
Yes	878	72.8	216	27.3	
No	487	27.2	76	16.4	

*General health*					
Ex./v. good	687	47.2	124	22.5	
Good	523	30.6	85	19.2	
Fair/poor	442	22.3	83	20.1	

*Physical activity*					
3 times/week	707	44.6	124	20.7	
LT 3 times/week	941	55.2	168	21.3	

*Region*					
Northeast	280	17.0	49	25.0	
Midwest	369	23.7	67	20.0	
South	565	35.4	85	18.4	
West	438	23.9	91	23.0	

*Cardiovascular risk groups*					*∗*
No risk	581	39.1	116	25.5	
One	510	29.0	78	16.4	
Two	343	20.3	66	22.3	
Three or more	218	11.5	32	14.9	

*Cardiovascular risk groups*					*∗∗*
No risk	581	39.1	116	25.5	
Yes	1,071	60.9	176	18.1	

Note: based on 1,652 adults aged between 22 and 64 years with migraine headache and alive during the calendar year. Cardiovascular risk factors consisted of the presence of any of the following conditions: diabetes, hypertension, hyperlipidemia, obesity, and smoking.

Asterisks represent significant group differences by triptans use based on chi-square tests.

CV: cardiovascular; GT: greater than; LT: less than; Rx: prescription; Wt.: weighted.

^*∗∗∗*^ *P* < 0.001; 0.001 ≤ ^*∗∗*^ *P* < 0.01; 0.01 ≤ ^*∗*^ *P* < 0.05.

**Table 2:** Adjusted odds ratios and 95% confidence intervals of cardiovascular risk factor from logistic regression on triptans use adults with migraine headache Medical Expenditure Panel Survey, 2009–2013.

	AOR	95% CI	Sig.
*CV risk factors group*			
No CV risk factors *(reference group*)			
One	0.55	[0.35, 0.86]	*∗∗*
Two	0.69	[0.43, 1.13]	
Three or more	0.36	[0.20, 0.66]	*∗∗∗*

*Reference group = one CV risk factor*			
*CV risk factors group*			
No CV risk factors	1.83	[1.17, 2.87]	*∗∗*
One *(reference group)*			
Two	1.27	[0.74, 2.17]	
Three or more	0.66	[0.35, 1.25]	

Note: based on 1,652 adults aged between 22 and 64 years with migraine headache and alive during the calendar year. Cardiovascular risk factors consisted of the presence of any of the following conditions: diabetes, hypertension, hyperlipidemia, obesity, and smoking.

Asterisks represent significant group differences in triptans use by cardiovascular risk factors based on logistic regression. The regressions controlled for sex, race/ethnicity, age, marital status, education, employment, poverty status, health insurance coverage, prescription insurance coverage, health status, physical activity, and region.

CV: cardiovascular; AOR: adjusted odds ratio; CI: confidence interval; sig: significance.

^*∗∗∗*^ *P* < 0.001; 0.001 ≤ ^*∗∗*^ *P* < 0.01; 0.01 ≤ ^*∗*^ *P* < 0.05.
